# Striving for Excellence: Fabrication of Complete Denture in a Patient With Flabby Ridges Using Specialized Impression Technique

**DOI:** 10.7759/cureus.62976

**Published:** 2024-06-23

**Authors:** Samiksha A Bute, Akansha Bansod, Arushi Beri

**Affiliations:** 1 Prosthodontics, Sharad Pawar Dental College and Hospital, Datta Meghe Instute of Higher Education and Research, Wardha, IND

**Keywords:** prosthodontics, hobkirk method, flabby tissue, specialized impression technique, complete denture

## Abstract

Tissue growth across the ridges is a typical clinical feature in the mandibular and maxillary arches. This excess tissue is known as flabby ridges. The mobile tissue may be distorted throughout the impression-making process due to the forces applied. The chewing forces will displace the denture if it is not adequately maintained, which will eventually cause the denture to lose its stability, support, and retention. The particular impression technique promoted accurately documenting flabby ridges. Several strategies, including implant therapy, balanced occlusal load distribution, surgical management, and special impression techniques, can be used to treat removable dentures with "flabby ridges."

This case study demonstrates the method for constructing a complete denture in a patient with flabby ridges using a specialized impression technique. This impression technique helps to record flabby tissue with minimal displacement, improving the stability, support, and retention of complete dentures.

## Introduction

A fibrous or flabby ridge is a movable region of soft tissue that exists superficial to the alveolar ridges of the maxilla as well as the mandible [1]. It is a highly movable or displaceable tissue that forms mostly when an edentulous ridge opposes the natural teeth. Hyperplastic soft tissue replacing alveolar bone results in the formation of flabby or movable ridges. These tissues are readily displaced and can negatively impact the stability, retention, and support of complete dentures. Despite the underlying movable tissue, these flabby ridges need to be controlled with specific, key approaches to provide a secure denture foundation. Approximately 24% of instances are seen in the edentulous maxilla, while only 5% occur in the edentulous mandible [2,3].

In the words of Kelly, extensive maxillary and mandibular ridge resorption along with alveolar bone loss can cause the "flabby ridge," a moveable tissue component found mainly on rugae as well as the tuberosity region in the maxilla, and also the anterior crest of the ridge alongside the retromolar area in the mandibular denture. Previously, flabby ridges in the anterior maxilla were associated with the "combination syndrome." The flabby ridge was assumed to occur due to a maxillary denture opposing mandibular anterior natural teeth without sufficient post-occlusal support [4]. Over time, denture users typically develop this kind of soft tissue, which can cause discomfort and ill-fitting dentures [5-7]. Considering flabby ridges and dentures, it is found that these ridges provide poor support for the denture. When using a denture on a flabby ridge, it is important to follow the right care and technique from the start to provide comfort and convenience. A flabby ridge must always be noted by the clinician [8]. Considering tissue displaceability, flabby tissues are divided into three groups based on their displaceability: low, average, and high. The average displaceability is clinically acceptable [9].

The problem with flabby ridges is that, due to the ridges being compressed during the impression-taking process, they are subjected to rebound and dislodge the denture underneath. As a result, an impression technique that compresses the non-flabby tissues is essential to offer optimal support while preventing flabby tissue displacement. These days easier-to-use polymers, like silicones, or polyvinylsiloxanes (PVS), are employed. Clinicians choose PVS materials because of their accuracy, and they are available in various viscosities that are suited for mucostatic and muco-compressive flabby ridge impressions. This modified impression technique differs from the standard one in that it is primarily performed by expert prosthodontists, is technique-sensitive, and provides good results. This article presents a case study utilizing the PVS impression material and an alternative impression procedure as reported by Hobkrik [10,11].

## Case presentation

**Figure 1:**
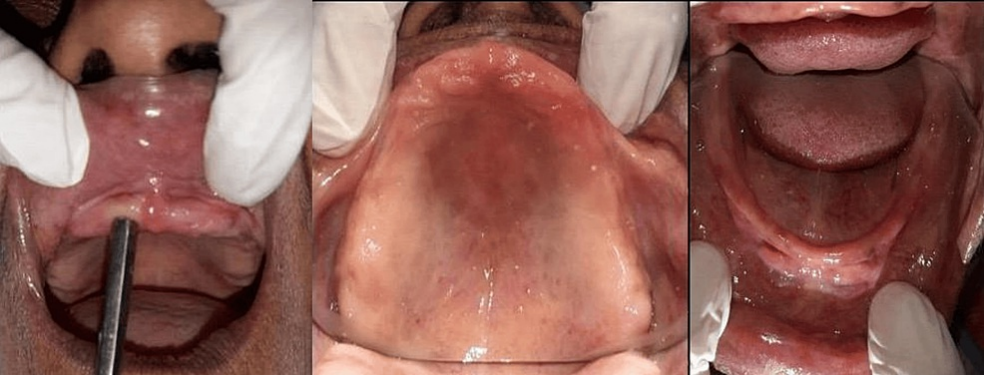
Flabby tissue present in the maxillary and the mandibular anterior region

A female patient, 56 years old, came to the Department of Prosthodontics, Crown and Bridge at Sharad Pawar Dental College and Hospital for prosthodontic rehabilitation. She complained that her previous denture was "loose" and that she had been without teeth since she was 54 years old. The intraoral examination showed the patient was completely edentulous and had a maxillary and mandibular arch with a markedly displaced anterior flabby ridge (Figure 1).

Despite the patient being advised of other treatment options, she was not prepared to have any surgery, either ridge augmentation or implant placement for the flabby ridge. In the end, the patient was told to proceed with a complete denture using a specialized impression procedure. The patient was slated to receive new complete dentures, and the Hobkirk method was employed to record flabby tissue in its undisplaced state. Before starting the procedure of fabrication of a new denture, the patient is advised to discontinue the use of the old denture and finger massaging for a week to achieve tissue rest.

For the maxillary ridge

**Figure 2:**
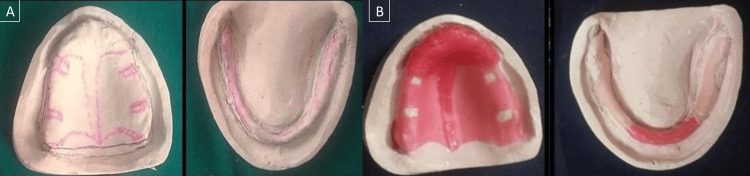
(A) Primary cast and (B) the double spacer is adapted to flabby tissue region

**Figure 3:**
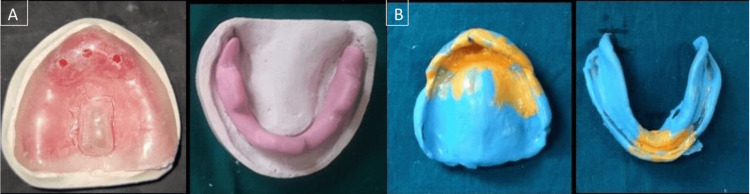
(A) Custom tray fabrication and relief holes were drilled and (B) the final impression was recorded using the Hobkirk technique

Irreversible hydrocolloid (Zhermack Dust-free Thixotropic Tropicalgin, Zhermack SpA, Badia Polesine (RO), Italy) was used to make a preliminary impression in the perforated edentulous tray, after which the primary cast was poured. A special tray was made using a full wax spacer, which was used on the cast. A single spacer was used over the remaining areas, such as the mid-palatine raphe, and a double spacer was applied to the area of the cast that had the flabby ridge to prevent displacement of flabby tissue (Figure 2). A custom tray was fabricated using the dough method over the spacer using self-cured acrylic resin. Borders were marked and trimmed 2 mm above the vestibule. A handle was made for easy stabilization. After that, a green stick impression compound, DPI - pinnacle impression compound, and tracing sticks (the Bombay Burmah Trading Corporation, Ltd., Mumbai, India), were used to complete border molding in a traditional manner. After scraping off the spacer wax, the final impression was made with medium-body elastomeric impression material (chromaclone™ PVS Putty - Ultradent, Germany). The impression material in the area of flabby tissue was then removed using a scalpel after the tray had been taken out of the mouth. To record flabby tissue, relief holes were drilled, and a tray containing wash-type or light-body elastomeric impression material was put in this location (Figure 3).

For the mandibular ridge

Irreversible hydrocolloid was used to make the preliminary impression in a perforated edentulous tray, and type II dental plaster was used to pour the primary cast (Figure 2).

The wax spacer is adapted on the primary cast with the exception of the posterior palatal seal region in the maxillary arch and the buccal shelf in the mandibular arch. Tissue stops are put in the canine as well as the first molar regions. Custom tray made from self-curing acrylic material. For border molding, green stick modeling material is used. The spacer wax was then scraped off, medium-body elastomeric impression material was used to get the final impression, and dental stone was used to make the master cast (Figure 3).

**Figure 4:**
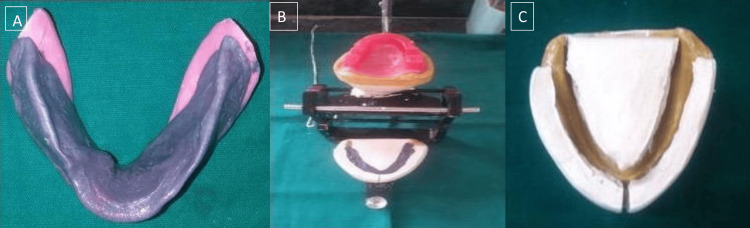
(A) Recorded neutral zone, (B) mounting, and (C) type II dental plaster matrices were created

Record bases are made in self-cure acrylic resin and wax occlusion rims are made. Jaw relations were recorded. On the second record base, a rim shape was created using mixed material that was made up of low- and medium-fusing impression compounds in a 3:7 ratio. After placement, the patient was instructed to talk and swallow in order to bring about a sufficient contraction of the muscles. All the actions were performed clearly and vigorously. Consequently, the form of the neutral zone was strengthened. To maintain the neutral zone on the cast, type II dental plaster matrices were created (Figure 4).

**Figure 5:**
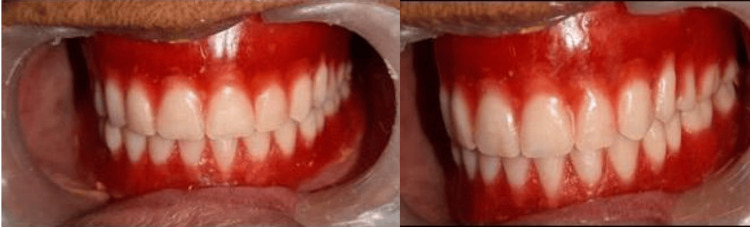
Trying the dentures

The wax rim was formed in a space confined by type II dental matrices, and it perfectly matched the neutral zone on newly fabricated baseplates on the mandibular cast. The artificial teeth setting was done within the matrices. The dentures were fabricated and try-in was done, and on insertion, the dentures had good retention and stability (Figure 5).

**Figure 6:**
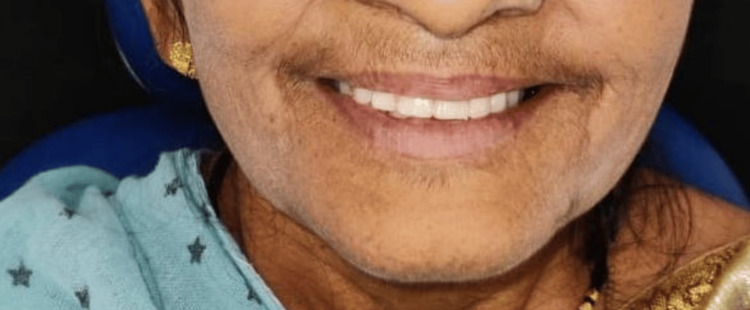
Satisfied patient with newly made complete denture using Hobkirk method

After laboratory procedures (acrylization, finishing, and polishing), the final denture was delivered (Figure 6) and post-insertion instructions were given. Follow-ups have been conducted at 24 hours, one week, one month, and six months.

## Discussion

For a complete denture to perform well, retention, stability, and support are fundamental concepts. When captured using any traditional technique, flabby tissues get squeezed in the middle of the impression. To achieve the best support, an imprint technique that compresses the non-flabby tissues without dislodging the flabby tissues is needed. Denture retention fails and the chance of dislocation increases due to the elastic rebound of soft fibrous tissue during function [12]. Impression technique is the key to carefully managing flabby ridges.

There are several possibilities for managing a flabby ridge: a conservative strategy or rehabilitation program, a prosthetic strategy, and a surgical strategy [13,14]. The choice of treatment is determined by the overall health of the patient, the clinical state of residual alveolar ridges, the financial circumstances of the patient, and the ability of the clinician. Conservative strategy to manage the flabby tissue includes tissue rest: before beginning the appropriate therapy, the prosthesis should be taken out of the mouth for a few days, for at least eight hours per day; soft tissue massage: for increased blood flow, the patient ought to massage their soft tissues at least twice or three times every day, and half a teaspoon of table salt diluted in a half glass of warm water can be used as mouthwash, therefore, instruct the patient to rinse; denture modification through flange and occlusal adjustment: use pressure-indicating paste (PIP) to identify and eliminate any uncomfortable places or pressure locations, and occlusal disharmonies are corrected through clinical remounting and vertical dimension restoration (VDO); tissue conditioning: soft tissue conditioners should be used to reline the old prosthesis before creating new dentures, and by acting as a cushion, absorbing occlusal loads, and dispersing them more equally among the supporting tissues, the tissue softener promotes mucosal healing. Every 72 hours, it must be changed [15]. If conservative therapy fails to alleviate the condition then a prosthetic method may be adopted, which includes impression procedures, the centric occlusal record, the occlusal shape, and the posterior tooth arrangement. The surgical approach includes surgical removal of flabby tissue followed by implant [16].

The literature has reported a variety of impression techniques and methods for recording flabby tissue throughout the impression-making process. Nevertheless, there is no proof that a particular impression technique, as opposed to others, will result in a denture that is stable and retains its shape on flabby ridges [17].

The Hobkrik method is presented in this study. This method makes use of a single, customized tray. The secondary impression is recorded using the heavy-bodied addition silicone. Relief holes are created, and the moveable tissue sections are removed. The wash impression is recorded using light-body impression material. Here, a light-body PVS impression material has been used to record the shape of the flabby ridge without displacing it [18]. Using different and distinctive techniques at each step, rather than regular procedures, will improve the quality of the complete denture fabrication. Conventional impression procedures for recording flabby tissues frequently result in the displacement of fibrous tissue, which then returns to its anatomical location, causing displacement of the final denture prosthesis. Thus, in the fabrication of complete dentures with flabby tissue, many prosthodontists recommended the practical application of this Hobkrik approach [19]. The only limitation of the technique is that it is static and hence multiple relief holes are made.

## Conclusions

The basic objectives of full-mouth rehabilitation should be met in terms of stability, retention, support, esthetics, and tissue preservation. It is critical to choose a technique and approach that will work for the operator as well as appropriately address the condition of the patient. Selective pressure or minimally displacive impression techniques work best when combined with modified impression procedures because they get around some of these limits and enable patients to receive dentures with good retention as well as stability without necessitating further appointments to the clinic. This is in contrast to normal conventional dentures. The management of flabby ridges can be a tedious task. Light-body impression materials for flabby ridges produce minimum tissue displacement; however, their uniform and controlled application is dependent on the operator's technique. This paper presents a modified impression technique that effectively applies light-body PVS impression material to create a non-displacing final impression of the flabby ridge. This modified impression technique allows for the successful management of ridges without additional clinical visits. Better retention and overall satisfaction are seen in dentures manufactured using the modified impression technique.
